# Hemoglobin level and lipoprotein particle size

**DOI:** 10.1186/s12944-018-0655-2

**Published:** 2018-01-10

**Authors:** Päivi Hämäläinen, Juha Saltevo, Hannu Kautiainen, Pekka Mäntyselkä, Mauno Vanhala

**Affiliations:** 1 Department of Internal Medicine, Tampere University Hospital, Teiskontie 35, 33521 Tampere, Finland; 2 Department of Medicine, Central Finland Central Hospital, Jyväskylä, Finland; 3 Unit of Family Practice, Central Finland Central Hospital, Jyväskylä, Finland; 4 Unit of Primary Health Care, Kuopio University Hospital, Kuopio, Finland; 5 Unit of Primary Health Care, University of Eastern Finland, and Kuopio University Hospital, Kuopio, Finland; 6 University of Eastern Finland and Kuopio University Hospital, Kuopio, Finland

**Keywords:** Liporotein particle size, VLDL, LDL, HDL, Hemoglobin

## Abstract

**Background:**

Alterations in lipoprotein size are associated with increased cardiovascular disease risk. Higher hemoglobin levels may indicate a higher risk of atherosclerosis and was previously associated with obesity, metabolic syndrome, and insulin resistance. No previous studies have investigated an association between hemoglobin concentration and lipoprotein particle size.

**Methods:**

We conducted a population-based, cross-sectional study of 766 Caucasian, middle-aged subjects (341 men and 425 women) born in Pieksämäki, Finland, who were categorized into five age groups. The concentrations and sizes of lipoprotein subclass particles were analyzed by high-throughput nuclear magnetic resonance (NMR) spectroscopy.

**Results:**

Larger very low density lipoprotein (VLDL) particle diameter was associated with higher hemoglobin concentrations in men (*p* = 0.003). There was a strong relationship between smaller high density lipoprotein (HDL) particle size and higher hemoglobin concentration in both men and women as well as with smaller low density lipoprotein (LDL) particle size and higher hemoglobin concentration in men and women (*p* < 0.001; *p* = 0.009, *p* = 0.008). VLDL particle concentration had a moderate positive correlation with hemoglobin concentration (*r* = 0.15; *p* < 0.001). LDL particle concentration showed a statistical trend suggesting increasing particle concentration with increasing hemoglobin levels (*r* = 0.08; *p* = 0.05).

**Conclusion:**

Higher hemoglobin levels are associated with larger VLDL, smaller LDL, and smaller HDL particle sizes and increasing amounts of larger VLDL and smaller LDL particles. This suggests that a higher hemoglobin concentration is associated with an unfavorable lipoprotein particle profile that is part of states that increase cardiovascular disease risk like diabetes and metabolic syndrome.

## Background

Lipoproteins consist of heterogeneous particles that differ in size. Alterations in lipoprotein sizes are associated with increased cardiovascular disease (CVD) risk [1–4] as well as in patients with conditions in which CVD risk is high like diabetes and metabolic syndrome [5–9]. Higher hemoglobin levels may indicate a higher risk of atherosclerosis [10] and was previously associated with obesity, metabolic syndrome, and insulin resistance [10–16]. However, as far as we are aware, no previous studies have investigated an association between hemoglobin concentration and lipoprotein particle size. Therefore, we conducted a cross-sectional study investigating any association between hemoglobin level and lipoprotein particle size assessed by proton nuclear magnetic resonance (NMR) spectroscopy.

## Methods

### Study subjects

The study population primarily consisted of 1294 middle-aged subjects from Pieksämäki, Finland, who were born in 1942, 1947, 1952, or 1962. These subjects were invited to a health check-up in the years 1997–1998 initially, and to a follow-up check-up in 2003–2004. A total of 766 subjects participated in the second health check-up in 2003–2004 when the hematological laboratory tests were performed. The final analysis included data from these 766 subjects. The study protocol was approved by the Ethics Committee of Kuopio University Hospital and the University of Eastern Finland. All participants provided informed written consent.

### Clinical and laboratory procedures

Both health check-ups were performed by the same two nurses. Waist circumference was measured and body mass index (BMI) was calculated. Current use of alcohol was considered low if the subject used no alcohol, moderate if the subject used less than two units per day, and high if the subject used more than two units per day. Physical activity was considered low if the subject exercised less than 30 min fewer than 3 times per week, moderate if the subject exercised at least 3 times per week, and high if the subject exercised every day.

Fresh blood samples were taken after an overnight fast. Plasma glucose concentration was measured using an automated colorimetric method (Peridochrom Glucose GOD-PAP, Boehringer, Germany). Plasma triglycerides were measured from fresh serum samples using enzymatic colorimetric methods (CHOD-PAP, GPO-PAP, Boehringer Mannheim GmbH, Germany). Plasma high density lipoprotein (HDL) cholesterol was measured using the same method after precipitation of low-density lipoprotein (LDL) cholesterol and very low-density lipoprotein (VLDL) cholesterol with phosphotungstic acid and magnesium. High-sensitivity C-reactive protein (hs-CRP) was measured with an Immunolite analyzer and a DPC hs-CRP assay (DPL, Los Angeles, CA, USA). Hemoglobin was measured using an automatic electronic cell calculator. All laboratory tests were analyzed at the Kuopio University laboratory during the years 2009–2010.

Concentrations and sizes of lipoprotein subclass particles were analyzed with high-throughput NMR spectroscopy of native serum samples [17, 18] in 2009. NMR data were measured at 37 °C using a Bruker AVANCE III spectrometer operating at 500.36 MHz using a new automated platform, as described previously [19]. The following 14 lipoprotein subclasses were calibrated using high-performance liquid chromatography: chylomicrons (CMs) and largest VLDL particles (CM/ largest VLDL; average particle diameter ± 75 nm); five different VLDL subclasses, i.e., very large (average particle diameter 64.0 nm), large (53.6 nm), medium (44.5 nm), small (36.8 nm) and very small VLDL (31.3 nm); intermediate-density lipoprotein (IDL; 28.6 nm); three LDL subclasses, i.e., large (25.5 nm), medium (23.0 nm), and small LDL (18.7 nm); and four HDL subclasses, i.e., very large (14.3 nm), large (12.1 nm), medium (10.9 nm), and small HDL (8.7 nm).

### Statistical methods

The data are presented as means and standard deviations. The 95% confidence intervals for the lipoprotein particle concentrations (and diameters) were obtained by bias-corrected, accelerated bootstrapping. Associations between the serum triglyceride, HDL, and total cholesterol concentrations with the NMR-measured concentrations were estimated with regression analysis using Sidak-adjusted probabilities. Multiple linear regression analysis was used to estimate the independent impacts of LDL, HDL, and VLDL particle diameter on the hemoglobin stratified by sex. In all hypotheses, *p* < 0.05 was considered significant.

## Results

Basic charasteristics of the study population are shown in Table 1*.* The study population included 425 women (55%) and 341 men with a mean age of 52.4 years and a mean body mass index (BMI) 27 kg/m^2^. Kidney function based on plasma creatinine level both in men and women was normal. Plasma alanine aminotransferase (alat) level was in normal female reference range (10–45 IU/L) in 99.5% of the women and in normal male reference range (10–70 IU/l) in 99.0% of men. Current smokers were 26% of men and 17% of women. Correlations between fasting plasma triglycerides, HDL, total cholesterol, and LDL, HDL and VLDL particle concentrations measured by NMR in women and men are shown in Table 2*.* Total plasma cholesterol had a high positive correlation with NMR-measured LDL particle concentration in both women and men (*r* = 0.92 and *r* = 0.89, respectively; *p* < 0.001). Also, plasma total cholesterol had a moderate positive correlation with NMR-measured VLDL concentration in women and men (*r* = 0.50 and *r* = 0.43, respectively; *p* < 0.001). Plasma triglycerides had a high positive correlation with the NMR-measured VLDL particle concentration in women (*r* = 0.85; *p* < 0.001) and a moderate positive correlation in men (*r* = 0.58; *p* < 0.001). Plasma HDL cholesterol had a high positive correlation with the NMR-measured HDL particle concentration in both women and men (*r* = 0.67 and *r* = 0.70, respectively; *p* < 0.001). There was no significant correlation between plasma triglycerides, HDL, or total cholesterol and NMR-measured LDL, HDL, or VLDL particle diameter (*Data not shown*)*.*

**Table 1:** Clinical and life-style characteristics of the study population

Characteristics	Men *N* = 341	Women *N* = 425	All *N* = 766
Age, years, mean (SD)	52.5 (6.2)	52.2 (6.5)	52.4 (6.4)
BMI, kg/m2, mean (SD)^a^	27.3 (3.9)	27.1 (5.0)	27.2 (4.6)
Waist, cm, mean (SD)	96.3 (11.3)	86.6 (12.2)	90.6 (12.8)
FP-glucose (mmol/L), mean (SD)^b^	6.1 (1.1)	5.8 (0.8)	5.9 (0.9)
Total cholesterol (mmol/L), mean (SD)	5.6 (0.9)	5.4 (1.0)	5.5 (1.0)
HDL-C (mmol/L), mean (SD)^c^	1.5 (0.4)	1.7 (0.3)	1.6 (0.3)
Triglycerides (mmol/L), mean (SD)	1.5 (1.0)	1.2 (0.5)	1.3 (0.7)
Hemoglobin, (g/L), mean (SD)	152.8 (9.2)	137.8 (9.0)	144.0 (11.7)
Hs-CRP (mg/L), mean (SD)^d^	1.8 (3.2)	2.1 (2.8)	2.0 (2.9)
Alat (I/U), mean (SD)	18.0 (10.9)	12.0 (7.6)	14.5 (9.6)
Creatinine (μmol/L), mean (SD)	87.1 (10.2)	75.8 (7.5)	80.1 (8.7)
Life-style factors, n (%)			
Current smoker	88 (26)	74 (17)	162 (21)
Current use of alcohol^e^			
Low (nothing)	46 (13)	92 (22)	138 (18)
Moderate	165 (49)	274 (64)	439 (57)
High	129 (38)	55 (13)	184 (24)
Physical activity n (%):			
Low	89 (26)	135 (32)	224 (29)
Moderate	193 (57)	238 (56)	431 (56)
High	59 (17)	49 (12)	108 (14)

Physical activity: Low = at least 30 min exercise less than 3 times/week, Moderate = at least 30 min exercise at least 3 times/week, High: e at least 30 min exercise daily

^a^BMI: Body mass index

^b^FP-glucose: fasting plasma glucose

^c^HDL-C: high density cholesterol

^d^Hs-CRP: high sensitivity C-reactive protein

^e^Current use of alcohol: Low = Nothing, Moderate = <2portions/day, High= >2 portions/day

**Table 2:** Correlations between plasma triglycerides, HDL or total cholesterol and NMR-measured LDL, HDL or VLDL particle concentration

Plasma cholesterol	NMR-measured particle concentrations		
	LDL r (95% CI)	HDL r (95% CI)	VLDL r (95% CI)
Women			
Triglycerides	0.29^a^	−0.07	0.85^a^
HDL	0.09	0.67^a^	−0.37^a^
Total	0.92^a^	0.25^a^	0.50^a^
Men			
Triglycerides	−0.01	−0.03	0.58^a^
HDL	0.16^b^	0.70^a^	−0.45^a^
Total	0.89^a^	0.34^a^	0.43^a^

Sidak-adjusted probabilities

^a^p < 0.001

^b^*p* < 0.05

**Fig. 1:**
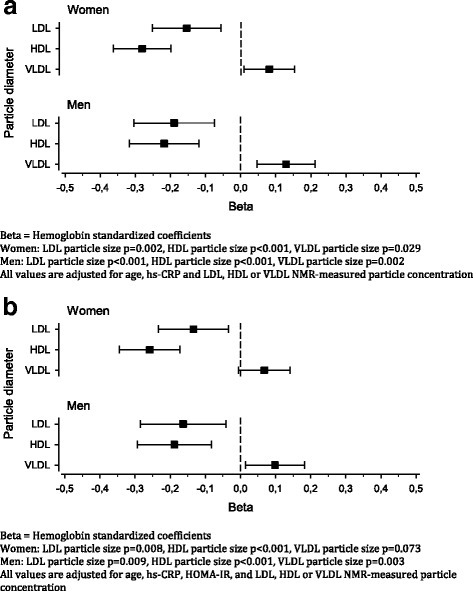
LDL, HDL and VLDL particle diameter changes in relation to hemoglobin concentration in women and men. **a** Beta = Hemoglobin standardized coefficients. Women: LDL particle size *p* = 0.002, HDL particle size *p* < 0.001, VLDL particle size *p* = 0.029. Men: LDL particle size *p* < 0.001, HDL particle size *p* < 0.001, VLDL particle size *p* = 0.002. All values are adjusted for age, hs-CRP and LDL, HDL or VLDL NMR-measured particle concentration. **b** Beta = Hemoglobin standardized coefficients. Women: LDL particle size *p* = 0.002, HDL particle size *p* < 0.001, VLDL particle size *p* = 0.029. Men: LDL particle size *p* < 0.001, HDL particle size *p* < 0.001, VLDL particle size *p* = 0.002. All values are adjusted for age, hs-CRP, HOMA-IR and LDL, HDL or VLDL NMR-measured particle concentration

**Fig. 2:**
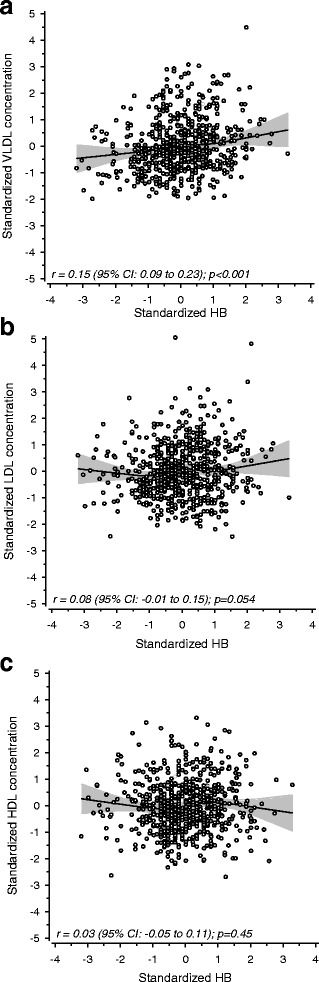
**a** Correlation between hemoglobin concentration and NMR measured VLDL particle concentration. **b** Correlation between hemoglobin concentration and NMR measured LDL particle concentration. **c** Correlation between hemoglobin concentration and NMR measured HDL particle concentration. All values are adjusted for gender, age and Hs-CRP

Figure 1 shows standardized coefficients (beta) between lipoprotein particle diameters and hemoglobin level. All values were first (Fig. 1a) adjusted for age, hs-CRP, and NMR-measured LDL, HDL, or VLDL concentrations. Larger VLDL particle diameter was associated with higher hemoglobin concentrations in both men and women (*p* = 0.002 and *p* = 0.029, respectively). There was a strong relationship between smaller HDL particle size and higher hemoglobin concentration in both men and women as well as lower LDL particle size and higher hemoglobin concentration in men (*p* < 0.001). Also, lower LDL particle size was associated with higher hemoglobin concentrations in women (*p* = 0.002). After adding adjusted-model the homeostasis model for assessment of insulin resistance (HOMA-IR) (Fig. 2b)*,* all results remain significant except larger VLDL diameter in women (*p* = 0.073). No significant association was found between serum ferritin level and the lipoproteins VLDL, LDL, or HDL particle diameter (data not shown).

Correlations between hemoglobin concentration and NMR-measured VLDL, LDL, and HDL particle concentration are shown in Fig. 2a-c. VLDL particle concentration had a moderate positive correlation with hemoglobin concentration (*r* = 0.15; *p* < 0.001). LDL particle concentration showed a statistical trend suggesting increasing particle concentration with increasing hemoglobin levels (*r* = 0.08; *p* = 0.05). There was no significant correlation between HDL particle concentration and hemoglobin.

## Discussion

In present study, we show an association between the particle size of the lipoproteins VLDL, HDL, and LDL and hemoglobin level. Although, associations between lipoprotein particle size and CVD, metabolic syndrome, obesity, insulin resistance, and type 2 diabetes have been investigated, we are not aware of previous studies reporting an association between hemoglobin concentration and lipoprotein particle size.

Larger VLDL particle size, smaller LDL particle size, and smaller HDL particle size were associated with higher hemoglobin concentrations. These associations remained unchanged after adjusting for concentrations of VLDL, LDL, or HDL particles. Previously, larger mean VLDL particle size was associated with impaired glucose tolerance, insulin resistance, and incidence of type 2 diabetes [5–9]. Also, previous studies showed an association between small LDL particle size and insulin resistance as well as incident diabetes, although the association with diabetes was not independent after adjusting for insulin sensitivity or triglycerides [8]. Additionally, small HDL particles were previously associated with reduced insulin sensitivity and hyperglycemia [7]. Our findings suggest that higher hemoglobin concentrations are associated with an unfavorable lipoprotein particle profile that is part of conditions such as metabolic syndrome and type 2 diabetes that increase CVD risk. Consequently, higher hemoglobin concentration can act as an additional marker indicating higher CVD risk profile.

Increasing VLDL particle concentration as well as increasing LDL particle concentration was associated with higher hemoglobin concentration, although the associations were weaker than with particle sizes. Consequently, higher hemoglobin level is associated with an increasing amount of larger VLDL and smaller LDL particles.

Previously, increasing hemoglobin level was associated with increasing arterial stiffness, which is used to assess CVD in high-risk populations [10]. Also, higher hemoglobin concentrations are present in individuals with metabolic syndrome or insulin resistance versus healthy controls or obese subjects versus non-obese subjects [11–16].

In hematological disorder polysytemia vera, elevated hemoglobin levels are associated with hypocholesterolemia and lower serum levels of total cholesterol and LDL cholesterol compared to subjects with elevated hemoglobin but without polysytemia vera [20]. Although studies investigating the association between elevated hemoglobin level and lipoprotein particle size in polysytemia vera patients (as far as we know) have not been done, hypocholesterolemia and lower LDL in polysytemia vera suggest different relation and mechanism than in our study.

The mechanisms that could explain the association between hemoglobin level and changes in lipoprotein particle size are unclear. Preliminary evidence of a relationship between hyperinsulinemia or insulin resistance and stimulated erythropoiesis exists [21]. Insulin can act as a growth factor for erythroid precursors [22]. Our results were also adjusted for HOMA-IR to evaluate the influence of insulin resistance. Adjusting did not change the results significance except the association of VLDL particle size and hemoglobin in women. This suggests that insulin resistance mostly affects the VLDL particles, but there exist also other mechanisms affecting the relation of lipoprotein particle size and hemoglobin level.

Hemoglobin level is influenced by iron status. We also investigated an association between serum ferritin level, as an indicator of iron stores, and lipoprotein particle size. Our results suggest that the association between hemoglobin and lipoprotein particle size is independent of serum ferritin levels and iron stores. Also, an association between lipoprotein particle size and hemoglobin level was significant after adjusting for hs-CRP level when excluding the influence of inflammation.

A limitation of our study is the cross-sectional design that does not allow us to identify proper causal relationships. However, the randomly selected, relatively large population, with no exclusion criteria is a strength of this study.

## Conclusions

In conclusion, higher hemoglobin levels are associated with larger VLDL, smaller LDL, and smaller HDL particle sizes and increasing amounts of larger VLDL and smaller LDL particles. This suggests that higher hemoglobin concentration is associated with an unfavorable lipoprotein particle profile that is part states that increase CVD risk like diabetes and metabolic syndrome.

## Abbreviations

BMI: Body mass index
CMs: Chylomicrons
CVD: Cardiovascular disease
HDL: High density lipoprotein
hs-CRP: High-sensitivity C-reactive protein
LDL: Low density lipoprotein
NMR: High-throughput nuclear magnetic resonance (spectroscopy)
VLDL: Very low density lipoprotein

## References

[1] RW MG, Craig DM, Haynes C (2016). High-density lipoprotein subclass measurements improve mortality risk prediction, discrimination and reclassification in a cardiac catheterization cohort. Atherosclerosis.

[2] Mora S, Szklo M, Otvos JD (2007). LDL particle subclasses, LDL particle size, and carotid atherosclerosis in the multi-ethnic study of atherosclerosis (MESA). Atherosclerosis.

[3] El Harchaoui K, van der Steeg WA, Stroes ES (2007). Value of low-density lipoprotein particle number and size as predictors of coronary artery disease in apparently healthy men and women: the EPIC-Norfolk prospective population study. J Am Coll Cardiol.

[4] Freedman D, Otvos J, Jeyarajah E (1998). Relation of lipoprotein subclasses as measured by proton nuclear magnetic resonance spectroscopy to coronary artery disease. Arterioscler Thromb Vasc Biol.

[5] Mora S, Otvos JD, Rosenson RS (2010). Lipoprotein particle size and concentration by nuclear magnetic resonance and incident type 2 diabetes in women. Diabetes.

[6] Lorenzo C, Hartnett S, Hanley AJ (2013). Impaired fasting glucose and impaired glucose tolerance have distinct lipoprotein and apolipoprotein changes: the insulin resistance atherosclerosis study. J Clin Endocrinol Metab.

[7] Wang J, Stančáková A, Soininen P (2012). Lipoprotein subclass profiles in individuals with varying degrees of glucose tolerance: a population-based study of 9399 Finnish men. J Intern Med.

[8] Mackey RH, Mora S, Bertoni AG (2015). Lipoprotein particles and incident type 2 diabetes in the multi-ethnic study of atherosclerosis. Diabetes Care.

[9] Jiang ZG, Boer HI, Mackey HR (2016). Associations of insulin resistance, inflammation and liver synthetic function with very low-density lipoprotein: the cardiovascular health study. Metabolism.

[10] Kawamoto R, Tabara Y, Kohara K (2012). A slightly low hemoglobin level is beneficially associated with arterial stiffness in Japanese community-dwelling women. Clin Exp Hypertens.

[11] Arakaki S, Maeshiro T, Hokama A (2016). Factors associated with visceral fat accumulation in the general population in Okinawa, Japan. World J Gastrointest Pharmacol Ther.

[12] Mansour M, Nassef YE, Shady MA (2016). Metabolic syndrome and cardiovascular risk factors in obese adolescent. Open Access Maced J Med Sci.

[13] Lohsoonthorn V, Jiamjarasrungsi W, Williams MA (2007). Association of hematological parameters with clustered components of metabolic syndrome among professional and office workers in Bangkok, Thailand. Diabetes Metab Syndr.

[14] Hämäläinen P, Saltevo J, Kautiainen H (2012). Erythropoietin, ferritin, haptoglobin, hemoglobin and transferrin receptor in metabolic syndrome: a case control study. Cardiovasc Diabetol.

[15] Laudisio A, Bandinelli S, Gemma A (2013). Metabolic syndrome and hemoglobin levels in elderly adults: the Invecchiare in Chianti study. J Am Geriatr Soc.

[16] Choi KM, Lee J, Kim YH (2003). Relation between insulin resistance and hematological parameters in elderly Koreans-Southwest Seoul (SWS) study. Koreans-Southwest Seoul (SWS) study. Diabetes Res Clin Pract.

[17] Ala-Korpela M (2008). Critical evaluation of 1H NMR metabonomics of serum as a methodology for disease risk assessment and diagnostics. Clin Chem Lab Med.

[18] Vehtari A, Makinen VP, Soininen P (2007). A novel Bayesian approach to quantify clinical variables and to determine their spectroscopic counterparts in 1HNMR metabonomic data. BMC Bioinformatics.

[19] Soininen P, Kangas AJ, Wurtz P (2009). High-throughput serum NMR metabonomics for cost-effective holistic studies on systemic metabolism. Analyst.

[20] Fujita H, Hamaki T, Handa N (2012). Hypocholesterolemia in patients with polycythemia vera. J Clin Exp Hematopathol.

[21] Barbieri M, Ragno E, Benvenuti E (2001). New aspects of the insulin resistance syndrome: impact on haematological parameters. Diabetologia.

[22] Miyagawa S, Kobayashi M, Konishi N (2000). Insulin and insulin-like growth factor I support the proliferation of erythroid progenitor cells in bone marrow through the sharing of receptors. Br J Haematol.

